# CO_2_ Conversion via Reverse Water Gas Shift Reaction Using Fully Selective Mo–P Multicomponent Catalysts

**DOI:** 10.1021/acs.iecr.2c00305

**Published:** 2022-08-19

**Authors:** Qi Zhang, Matthew Bown, Laura Pastor-Pérez, Melis S. Duyar, Tomas R. Reina

**Affiliations:** Department of Chemical and Process Engineering, University of Surrey, Guildford, GU2 7XH, United Kingdom

## Abstract

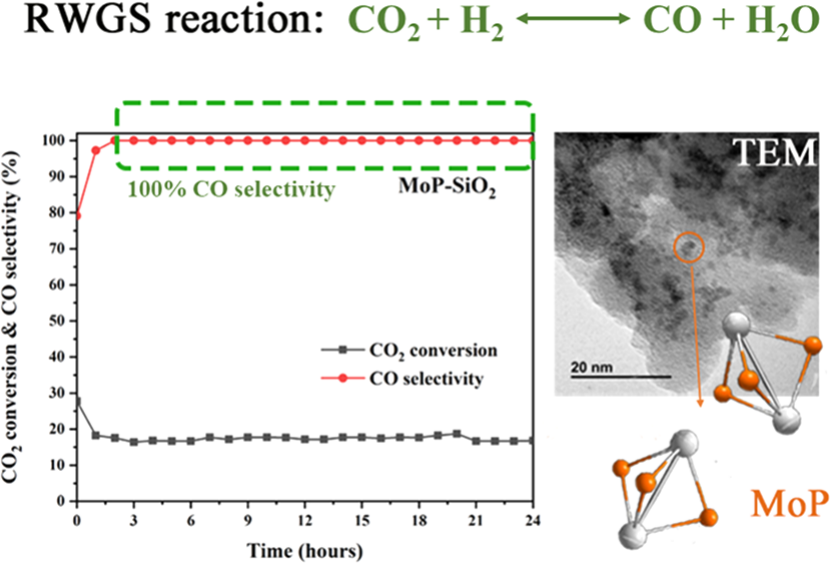

The reverse water gas shift reaction (RWGS) has attracted much attention as a potential means to widespread utilization of CO_2_ through the production of synthesis gas. However, for commercial implementation of RWGS at the scales needed to replace fossil feedstocks with CO_2_, new catalysts must be developed using earth abundant materials, and these catalysts must suppress the competing methanation reaction completely while maintaining stable performance at elevated temperatures and high conversions producing large quantities of water. Herein we identify molybdenum phosphide (MoP) as a nonprecious metal catalyst that satisfies these requirements. Supported MoP catalysts completely suppress methanation while undergoing minimal deactivation, opening up possibilities for their use in CO_2_ utilization.

## 1 Introduction

The global warming caused by excessive greenhouse gases (GHGs) has become one of the greatest environmental threats in the world. Among these different GHG emissions, such as water vapor, CH_4_, and CO_2_, CO_2_ is an important one which is mainly emitted from oil refineries, power plants, cement production, and steel and iron industries.^1^ Due to the greenhouse effect, several CO_2_ conversion technologies are proposed. Among the different CO_2_ upgrading processes, the reverse water gas shift (RWGS) reaction represents a viable route to convert CO_2_ and H_2_ into CO and water (eq 1), and the product CO could be used in downstream Fischer–Tropsch (FT) or MeOH synthesis processes.^2,3^ However, due to the endothermic nature of the process, the RWGS reaction requires high temperatures to achieve equilibrium CO_2_ conversions. In addition, the CO_2_ methanation is a side reaction (eq 2) which must be suppressed by using a selective catalyst. Therefore, considerable efforts have been made to develop thermally stable catalysts with high activities and selectivities toward carbon monoxide.^4^
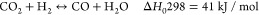
1
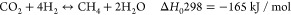
2Normally, the RWGS catalysts consist of well dispersed metal active sites on high surface area metal oxide supports.^5^ In terms of metal sites, copper^6^ and some noble metals (Pt,^7^ Pd,^8^ and Rh^9^) have been studied extensively. Concerning the support, CeO_2_ is one of the most widely used for the RWGS reaction because of its excellent redox properties.^6^ In addition to the metal oxide supports, transition metal carbides (TMCs) have been identified as desirable materials for the RWGS reaction as their properties are similar to Pt-group precious metals.^10^

Although transition metal phosphides (TMPs) have been investigated in the energy industry,^10−12^ in the past decades, the research dealing with TMPs catalysts for CO_2_ upgrading reactions are still relatively scarce compared to the materials listed above. Among the TMPs, molybdenum phosphide (MoP) catalysts exhibit stable performance toward methanol synthesis from CO_2_ and CO.^13,14^ During high pressure CO_2_ hydrogenation experiments for methanol synthesis, MoP catalysts have been observed to catalyze some CO formation as a byproduct.^13,14^ The molybdenum phosphide phase is theoretically expected to remain stable under hydrogenating conditions^13^ and has been shown experimentally to retain its chemical structure up to 950 °C in hydrogen,^15^ making it a suitable catalyst for the RWGS reaction. Our group has previously used a DFT-based mechanistic study to explore the potential activity of MoP (0001) for the RWGS reaction and found that this surface is an active phase for the RWGS reaction.^2^ This theoretical work and previously reported activity and stability of MoP for CO_2_ reduction leads us to investigate the performance of MoP catalysts toward the RWGS reaction experimentally.

Among these widely used metal oxide supports, the combination of MoP and SiO_2_ has already been shown to result in high activity for methanol synthesis from CO_2_.^13,14^ In addition, SiO_2_ shows the potential to prevent the agglomeration of metal sites leading to enhanced catalytic activity levels in the hydrogenation reactions. Al_2_O_3_ is also a widely investigated support for RWGS which could facilitate the dispersion of the active phase and boost oxygen mobility.^16,17^ However, the acidity of Al_2_O_3_ can induce coking. When seeking for a fair balance acid–base properties and coking mitigation solution, the addition of ceria to alumina-based supports could decrease the overall acidity thus helping to avoid carbon deposition due to enhanced oxygen mobility ascribed to CeO_2_-based systems.^18,19^ Herein we investigate a series of molybdenum phosphide catalysts supported on SiO_2_, Al_2_O_3_, and CeAl for the RWGS reaction.

## 2 Experimental Section

Experimental methods are summarized here, with more detailed descriptions available in the Supporting Information (SI).

### 2.1 Catalysts Preparation

Catalysts were synthesized using a wet impregnation method. Ammonium heptamolybdate [(NH_4_)_6_Mo_7_O_24_] (Sigma-Aldrich) and diammonium hydrogen phosphate [(NH_4_)_2_HPO_4_] (Sigma-Aldrich) were mixed to obtain a P/Mo atomic ratio of 1.2:1, as a slightly phosphorus rich synthesis was shown previously to be beneficial for the formation of the MoP phase.^20^ This mixture was dissolved in deionized water and added to the point of incipient wetness of the supports (Sigma-Aldrich). The weight loading of MoP was 15 wt % for all supports. The solution was dried in an oven for 12 h at 80 °C before calcining for 5 h at 500 °C. The precursor was reduced in a fixed bed reactor, where the sample was heated from room temperature to 650 °C using a ramp of 2 min^–1^ followed by holding at this temperature for 2 h. Reduction took place with a flow of 60 mL min^–1^ H_2_ before being cooled to room temperature in N_2_. The sample was passivated at room temperature in a flow of 40 mL min^–1^ of 1.5% O_2_/argon for 12 h. This method was repeated for each of the three selected supports: silica (SiO_2_, Sigma-Aldrich), alumina (Al_2_O_3_, Sigma-Aldrich), and ceria-alumina (CeO_2_–Al_2_O_3_, Sigma-Aldrich).

The catalysts prepared with different supports are referred as Mo–P–SiO_2_, Mo–P–CeAl, and Mo–P–Al_2_O_3_ in this manuscript.

### 2.2 Catalysts Characterization

X-ray diffraction (XRD), X-ray photoelectron spectroscopy (XPS), temperature-programmed oxidation (TPO), transmission electron microscopy (TEM), H_2_–temperature-programmed reduction (TPR) and BET surface area measurement are used in this work to characterize the prepared catalysts.

### 2.3 Catalytic Testing

The RWGS tests were evaluated within a temperature range of 400 to 750 °C at a constant weight hourly space velocity (WHSV) of 12 000 mL g^–1^ h^–1^ for all synthesized catalysts. Stability tests were conducted at a space velocity of 12 000 mL g^–1^ h^–1^ with a H_2_/CO_2_ ratio of 4:1 at 550 °C for 24 h. The continuous temperature-programmed RWGS reaction was conducted within a temperature range of 300 to 750 °C using the mass spectrum for product analysis at a space velocity of 12 000 mL g^–1^ h^–1^ with a H_2_/CO_2_ ratio of 4:1.

Performance of the catalysts are reported in terms of CO_2_ conversion (eq 3), CO selectivity (eq 4), and CH_4_ selectivity (eq 5). Where *n*_CO_2_in_ is the initial molar flow rate (kmol/min) of CO_2_ in the reactant mixture and *n*_COout_, *n*_CH_4_out_, and *n*_CO_2_out_ are the outlet molar flow rates in the product stream of CO, CH_4_, and CO_2_, respectively.
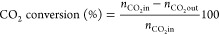
3
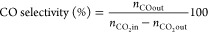
4
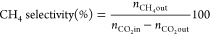
5

## 3 Results and Discussion

### 3.1 Characterization of as-Synthesized Catalysts

**Figure 1:**
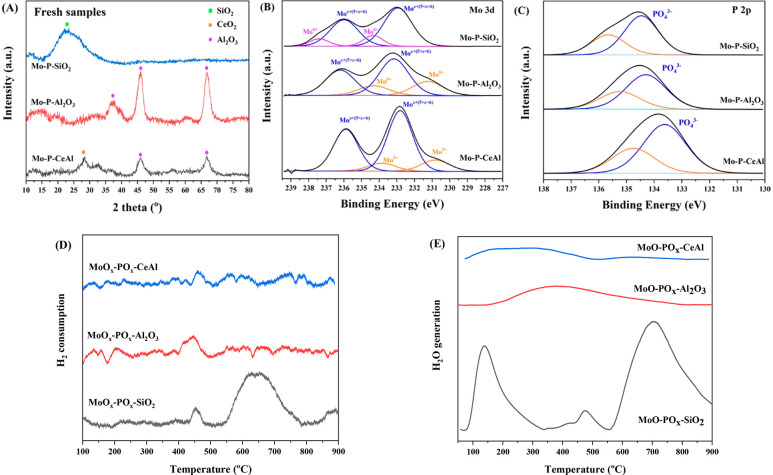
(A) X-ray diffraction patterns; (B) X-ray photoelectron spectroscopy Mo 3d spectra; (C) P 2p spectra and the deconvoluted peaks for fresh Mo–P–SiO_2_, Mo–P–Al_2_O_3_, and Mo–P–CeAl samples; (D,E) H_2_–temperature-programmed reduction (TPR) results for the precursors of Mo–P–SiO_2_, Mo–P–Al_2_O_3_, and Mo–P–CeAl.

Figure 1A displays the XRD pattern of the as-synthesized molybdenum phosphide catalysts. The crystalline MoP phase cannot be detected on any of the catalysts via XRD, indicating this phase is highly dispersed as nanoparticles, present as an amorphous phase or a mixture of well dispersed phases (phosphide and phosphate). For the Mo–P–SiO_2_ catalyst, the broad scattering maximum centered at 22.5° is ascribed to amorphous SiO_2_.^21,22^ For Mo–P–Al_2_O_3_ and Mo–P–CeAl catalysts, the peaks labeled by purple dots are assigned to γ-Al_2_O_3_ (JCPDS No. 29-0063).^23,24^ In addition, the peak at 2θ = 28.7° in the Mo–P–CeAl sample is attributed to the cubic fluorite-type CeO_2_ structure (JCPDS No. 81-0792).^25,26^ Molybdenum oxide peaks were not detected on any catalyst.

The surface chemistry and the electronic properties of these prepared samples were studied by XPS. Mo 3d and P 2p XPS spectra were collected (Figure 1B,C and Table 1). Mo 3d spectra are split into 3d_5/2_ and 3d_3/2_ peaks due to the spin–orbital coupling effect.^27^ For the Mo–P–Al_2_O_3_ and Mo–P–CeAl catalysts, it is found that there are two different Mo valence states species on the surface. The one with Mo 3d_5/2_ binding energy of 231 ± 0.3 eV is identified as Mo^5+^ species involved in Mo_2_O_5_.^28−31^ Doublets with Mo 3d_5/2_ peaks at 233 eV ± 0.2 eV should be assigned to Mo^ε+(V<ε<VI)^. For the Mo–P–SiO_2_ catalyst, the Mo 3d_5/2_ BE at 234.3 eV are characteristic of Mo^6+^ which suggests the presence of MoO_3_^28,30,32,33^ or Mo^6+^ in molybdenum phosphate.^34^ The P 2p scan is shown in Figure 1C. The peaks located around 134 eV can be ascribed to molybdenum phosphate species as a consequence of passivation.^35,36^

**Table 1:** Mo 3d_5/2_ and P 2p_3/2_ Binding Energies of All Samples

	Mo 3d_5/2_(eV)			P 2p_3/2_ (eV)	
sample	Mo^5+^	Mo^ε+(5<ε<6)^	Mo^6+^	P^5+^	P/Mo
Mo–P–SiO_2_		232.9 (86.7%)	234.3 (16.3%)	134.5	1.56
Mo–P–Al_2_O_3_	231.3 (28.6%)	233.2(71.4%)		134.3	1.41
Mo–P–CeAl	230.8 (17.6%)	232.8 (82.4%)		133.6	1.72

The XPS analysis results indicated that the surfaces of these synthesized catalysts have been fully oxidized, which is expected from the passivation and air exposure of the catalysts after synthesis. Peaks corresponding to MoP (which would be in the range 227.1–227.7 eV) could not be detected.^20^ We have previously shown via XAS and XRD that the MoP phase formation is heavily dependent on support-precursor interactions, and exposure to air results in surface oxidation which is reversed upon treatment in hydrogen.^13^ Our results are consistent with previous work reporting that MoP on silica cannot be observed at low loadings (<25 wt %) via XPS and XRD.^34^ In the presence of CeO_2_ on the Al_2_O_3_ support (Mo–P–CeAl), the binding energy of Mo shifts to a lower valence state than in Mo–P–Al_2_O_3_. Since CeO_2_ has excellent reducibility,^18,37,38^ we proposed that the n-type semiconductor property of CeO_2_ plays a key role in this process and promotes the reduction of surface phosphate to a larger extent.

To test this hypothesis and gather further understanding of catalysts’ reduction features and the interactions among the molybdenum phosphide/phosphate phases and the different supports, H_2_-TPR was conducted on the catalyst precursors (before reduction). Figure 1D,E shows hydrogen consumption and water generation profiles of the studied samples from room temperature to 900 °C. The precursor of Mo–P–SiO_2_ presents the typical reduction peak around 450 °C corresponding to the reduction of Mo^6+^ (MoO_3_) species to Mo^4+^ (MoO_2_). The maximum peak at 650 °C corresponds to the co-reductions of Mo^4+^ to Mo^0^ and of P^5+^ to P^0^. The water generation peak of Mo–P–SiO_2_ precursor matched well with the H_2_ consumption peak (Figure 1E), the extra peak located at ∼150 °C should be assigned to physically adsorbed water.^39^ For the precursor of alumina-containing Mo–P, molybdenum precursor should be reduced to the metallic state first and then react with P to form phosphide according to the previous report from the Oyama group.^40^ In this work, peaks around 450 °C were detected, consistent with some degree of MoO_*x*_ reduction. But there was no main peak detected while heating from 600 to 800 °C. It is likely due to the formation of aluminum phosphates; the reduction of aluminum phosphates are reported to occur at *T* > 850 °C.^40^ While all the phosphate is not reduced, an excess P (P/Mo = 1.2:1) ratio was used in our synthesis which is known to improve MoP formation.^20^ Therefore, while the XPS results leads us to believe better reducibility on CeAl, TPR shows this is a surface effect and the bulk reduction of the catalyst is not affected because of the different MoP formation mechanisms on alumina supported MoP due to the presence of aluminum phosphates. In addition, the TPR results suggest that MoP formation occurs on SiO_2_ supported catalysts at the temperature we employed in the synthesis, but on alumina supported catalysts the reduction of phosphates (likely bound to aluminum) is not complete.

The P/Mo ratio shown in Table 1 indicates that the surfaces of all prepared catalysts are rich in P. Although the P/Mo ratio used in synthesis is 1.2, all the composition values of P/Mo shown in Table 1 are higher than 1.4. A similar phenomenon has been observed in MoP-K-SiO_2_ catalysts. In that case, even though the synthesis P/Mo ratio was equal to 1.5, a P/Mo ratio higher than 2 was observed for all catalysts. The higher P/Mo ratio might be attributed to the formation of a P-rich phosphate shell over MoP that is later reduced to a P-rich MoP_*x*_ species.^13^

### 3.2 Catalytic Performance

**Figure 2:**
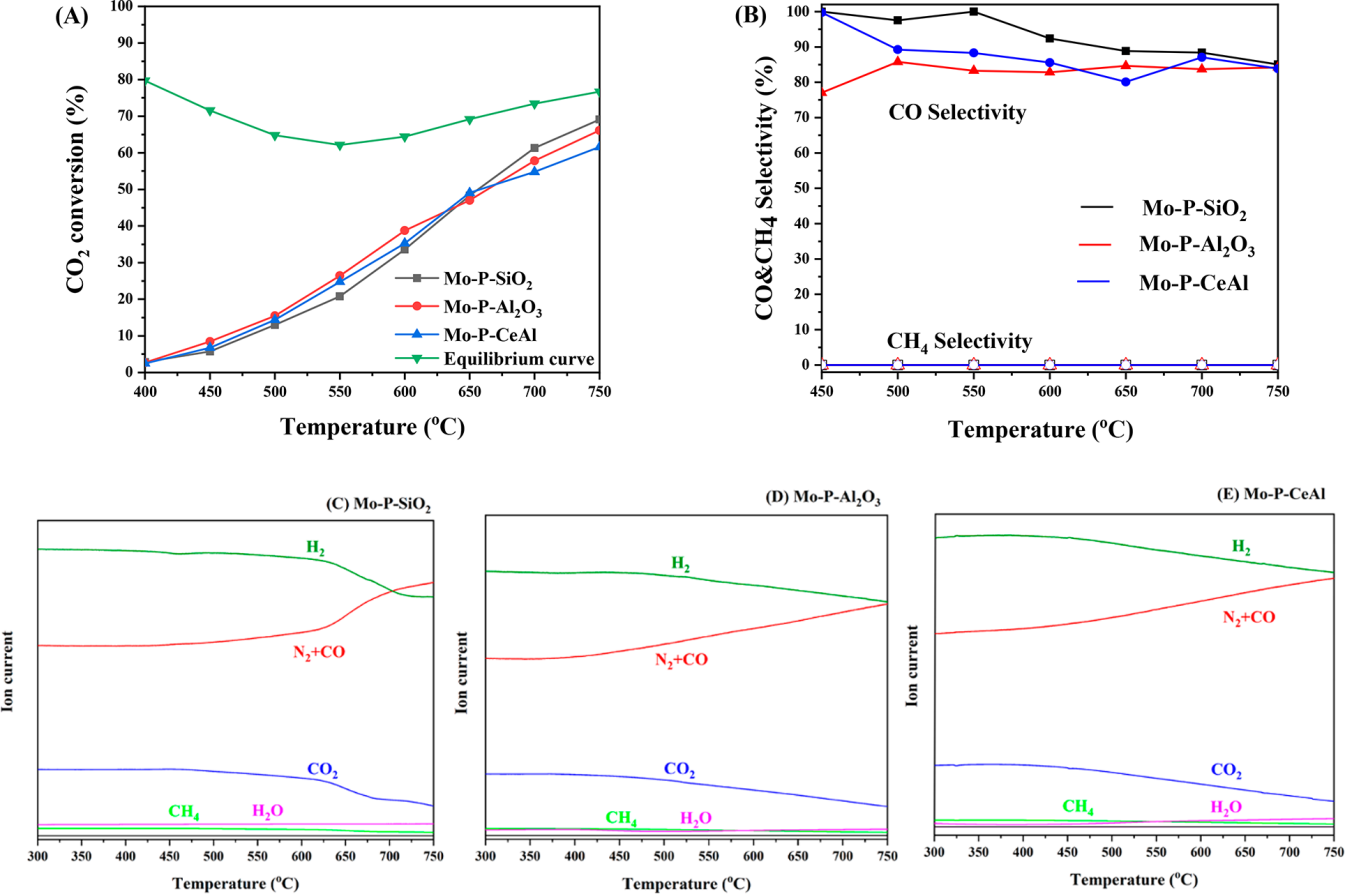
(A) CO_2_ conversion (B) CO and CH_4_ selectivity for Mo–P–SiO_2_, Mo–P–Al_2_O_3_, and Mo–P–CeAl. Mass spectrum for (C) Mo–P–SiO_2_, (D) Mo–P–Al_2_O_3_, and (E) Mo–P–CeAl. Condition: H_2_/CO_2_ = 4:1, WHSV = 12 000 mL g^–1^ h^–1^.

Figure 2A shows the CO_2_ conversion trends over the prepared catalysts as a function of temperature. The CO and CH_4_ selectivities are displayed in Figure 2 (B). All the synthesized catalysts are active for RWGS in the temperature range 400–750 °C and more importantly the Sabatier reaction is completely suppressed, despite the high H_2_/CO_2_ ratio used. Mo–P catalysts are highly selective toward RWGS at ambient pressure.

In terms of the CO_2_ conversion, the performance of Mo–P–SiO_2_ is slightly better than that of Mo–P–Al_2_O_3_ and Mo–P–CeAl in the high temperature range (650–750 °C). In the 450–600 °C range, the CO_2_ conversion toward Mo–P–Al_2_O_3_ shows the best CO_2_ activity than the other two. But in general, the performances of these three studied catalysts are similar.

All the synthesized catalysts exhibit high CO selectivity (>80%) in the temperature range 450–750 °C (Figure 2B). Mo–P–SiO_2_ is the most selective catalyst, especially in the temperature range of 450–550 °C, producing nearly 100% CO. As shown in the TPR section, the temperature we employed in the synthesis is suitable to produce silica-supported MoP, but for alumina-supported MoP catalysts, there are phosphates remaining on the surface under our synthesis condition, hence the different phosphorus compounds are likely to be the reason for different CO selectivity. In addition, our group has previously used systematic DFT (density functional theory) study on MoP (0001) to explore its potential for applications in chemical CO_2_ recycling via the RWGS reaction. Mechanistic investigation using potential energy surface (PES) profiles in this work showcased that MoP was active toward the RWGS reaction with the direct path (CO_2_* → CO* + O*) favorable on MoP (0001). Furthermore, it was observed that CH_4_* formation relative to CO* on the MoP (0001) surface requires higher energy from the PES profile thermodynamically, hence the MoP (0001) surface was more selective toward CO than CH_4_ generation.^2^ In our case, the Mo–P–SiO_2_ catalyst with more MoP present on the surface exhibited higher CO selectivity than alumina-supported Mo–P catalysts, consistent with the DFT calculation. Therefore, we attribute the high CO selectivity toward the Mo–P–SiO_2_ catalyst to the MoP phase generated on the surface of the SiO_2_ support.

As can be seen from Table 2, the carbon balance did not reach 100% toward the tested catalysts for most of the temperatures. For the Mo–P–SiO_2_ catalyst, the carbon balance is ∼100% in the 450–550 °C range and decreased gradually with increasing temperature. Since no methane was detected, this indicates that there are either other gas phase products (other than CO&CH_4_) and/or the deposition of carbon species on the catalysts.

**Table 2:**
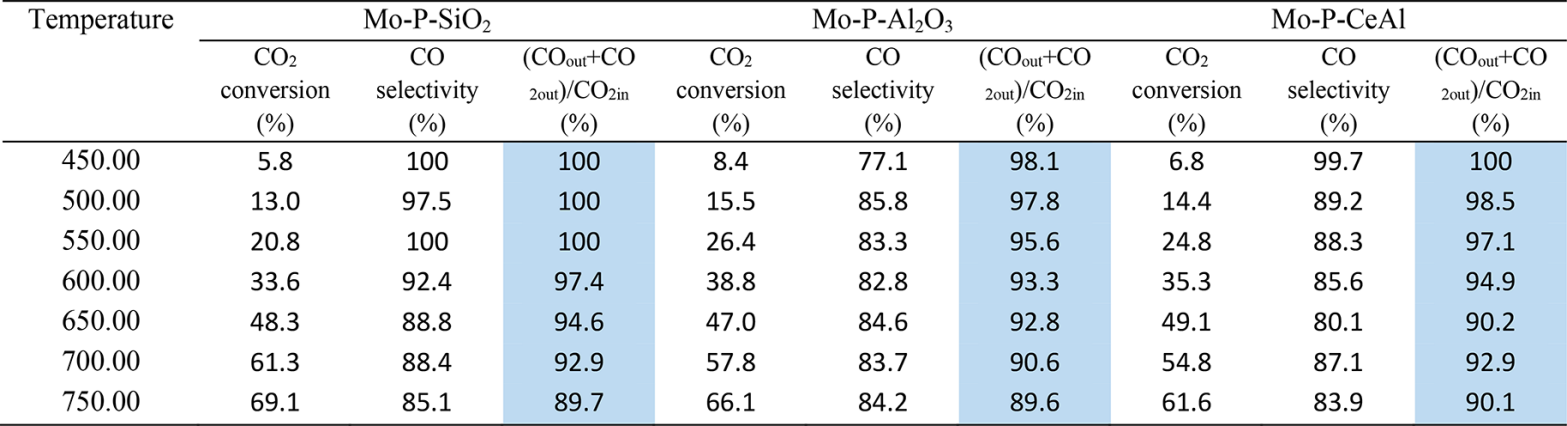
CO_2_ Conversion, CO Selectivity, and Carbon Balance Calculation toward Synthesized Catalysts

To measure if there are other gas phase species present, a continuous temperature-programmed RWGS reaction was conducted using the mass spectrum for product analysis. In our previous work, CH_4_, CO, and methanol as well as C_2+_ oxygenates and hydrocarbons were detected as gas phase products when MoP/Al_2_O_3_ and MoP/CeO_2_ were tested for CO_2_ hydrogenation reaction at 40 bar.^14^ Hence, we monitored C_2_H_4_, C_2_H_6_, CH_3_OH, and C_2_H_5_OH as possible products along with CH_4_ and CO. No change in ion current was detected for C_2_H_4_, C_2_H_6_, CH_3_OH, and C_2_H_5_OH. The signals for CO, CH_4_, H_2_O, CO_2_, and H_2_ are shown in Figure 2C,D,E and agree with our conversion and selectivity data shown in Figure 2A,B. This is indicative of the missing carbon being deposited as solid carbon on the catalysts. The carbon deposition is investigated further in the next section by temperature-programmed oxidation (TPO) and thermogravimetric analysis (TGA).

### 3.3 Post-reaction Characterization

**Figure 3:**
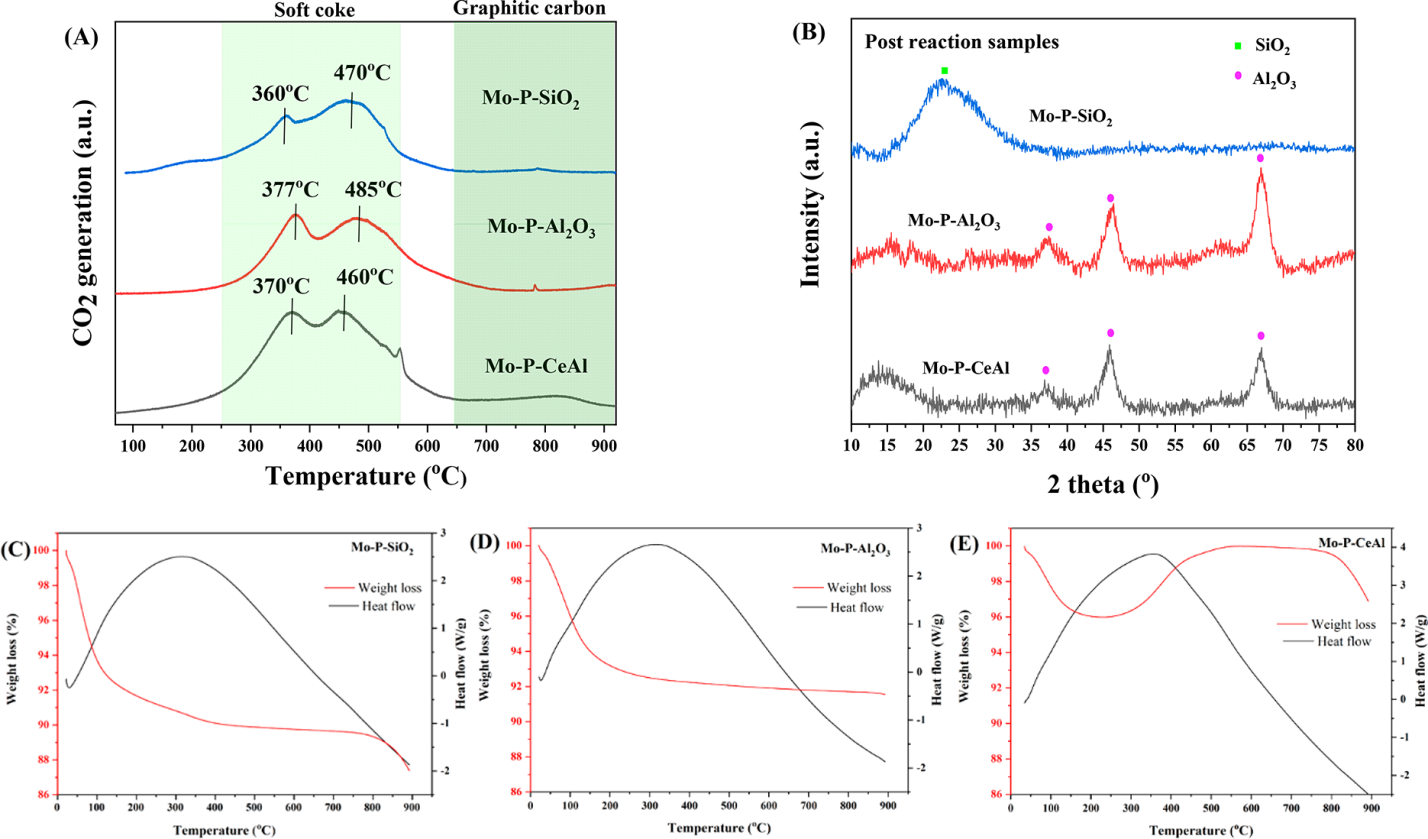
(A) Temperature-programmed oxidation (TPO); (B) X-ray diffraction patterns result for post-reaction Mo–P–SiO_2_, Mo–P–Al_2_O_3_, and Mo–P–CeAl; thermogravimetric analysis (TGA) for post-reaction (C) Mo–P–SiO_2_, (D) Mo–P–Al_2_O_3_, and (E) Mo–P–CeAl.

Figure 3 shows the TPO, XRD, and TGA results of the post-reaction samples. All the samples used in this section are post-temperature-screening samples that have been tested under RWGS reaction conditions (H_2_/CO_2_ = 4:1, WHSV = 12 000 mL g^–1^ h^–1^) from 400 to 750 °C, one hour for each temperature.

O_2_-TPO experiments of the post-reaction catalysts were carried out, and the results are shown in Figure 3A. Certain temperature ranges of CO_2_ peaks can be attributed to the different types of carbonaceous species. The peaks corresponding to the active intermediates in the RWGS reaction appeared lower than 380 °C.^41,42^ The second range peaks between 440 °C and 640 °C are assigned to whisker carbon formed on or close to Mo oxides.^43,44^ In general, the most refractory carbon is the graphitic carbon formed on the support (temperature range: TPO > 650 °C), which does not appear in these three catalysts.^44,45^ The first two fractions of coke were classified as soft coke which can be removed at lower temperatures, in this case, below 600 °C.^46^ As can be seen in the Figure 3A, the carbon deposited on Mo–P–SiO_2_ and Mo–P–CeAl can be more easily removed by treatment in hydrogen at mild conditions than on Mo–P–Al_2_O_3_. In addition, the TPO result confirms that carbon deposition happened during the RWGS reaction, which can explain the less than 100% carbon balance at certain temperatures.

Figure 3B displays the XRD patterns for post-RWGS reaction samples. All the SiO_2_ and Al_2_O_3_ peaks are observed in fresh samples (Figure 1A), with no new phases. Only crystalline CeO_2_ disappeared after the RWGS test in Mo–P–CeAl, indicating the reduction of CeO_2_ to an amorphous Ce (3+) species during the RWGS performance test.

In order to further quantify the carbon deposition, TGA tests were conducted for all the post-reaction samples. Generally speaking, most of the carbon combustion happens below 400 °C, and the heat flows show broad positive curves indicating an exothermic process, consistent with oxidation. For the Mo–P–SiO_2_ catalyst, it was observed that the weight loss caused by coking is 12.6% (Figure 3C), hence the carbon formation on the 250 mg catalyst is 36.1 mg. Based on the reaction conditions used in the RWGS test (5 mL/min inlet CO_2_ flow, 1 h test for each temperature) and the catalytic performance shown in Figure 2, the missing carbon during the performance test is 48.3 mg (the detailed calculation can be seen in the SI). Therefore, around 75% of the missing carbon became the coke formation deposited on the surface of the Mo–P–SiO_2_ catalysts. For the Mo–P–Al_2_O_3_ catalyst, the weight loss caused by coking is around 8.5% (Figure 3D) and the corresponding carbon formation is 23.1 mg. However, the missing carbon during the RWGS test toward Mo–P–Al_2_O_3_ is around 66.3 mg, indicating that there are some other gas phase products have not been detected. For the Mo–P–CeAl catalyst the plot trend is different than for the other two catalysts (Figure 3E). The weight decreased at the beginning, but when the temperature reached 300 °C, it started to increase. The first decrease should be attributed to the carbon combustion like that for the other two catalysts, the further mass increase could be assigned to the oxidation of the CeO_*x*_ phase. As can be seen in the post-reaction XRD pattern, crystalline CeO_2_ disappeared in Mo–P–CeAl after the RWGS test, indicating that the reduction of CeO_2_ happened during the RWGS reaction. Here the amorphous Ce^3+^ species might have been fully oxidized to CeO_2_ again during the TGA test; hence, a 4% weight gain shows in the TGA plot. The TPO results showcase that the carbon deposition is not the determining factor of the catalytic performance, despite the higher amount of carbon deposition on Mo–P–SiO_2_, it still shows higher CO selectivity than Mo–P–Al_2_O_3_. Since MoP is proposed to be very selective toward CO generation in our previous theoretical study,^2^ the greater presence of MoP on the surface of Mo–P–SiO_2_ is likely to be the reason for the CO selectivity difference.

### 3.4 Stability Test

Since all three catalysts exhibit similar CO_2_ conversions, the one showing the best CO selectivity (Mo–P–SiO_2_) was chosen to assess 24 h stability during the RWGS. Normally, the RWGS reaction is combined with a Fischer–Tropsch process aiming for an integrated process of CO_2_ to fuels. The Fischer–Tropsch process is generally operated in the temperature range of 150–300 °C, while the endothermic nature of the RWGS imposes high operational temperatures. In this sense, the successful implementation of a medium/low-temperature RWGS catalyst would represent a step ahead in this technology, facilitating energy and process integration. Thus, 550 °C was selected as reaction temperature in here to bridge the RWGS-FTS gap.

**Figure 4:**
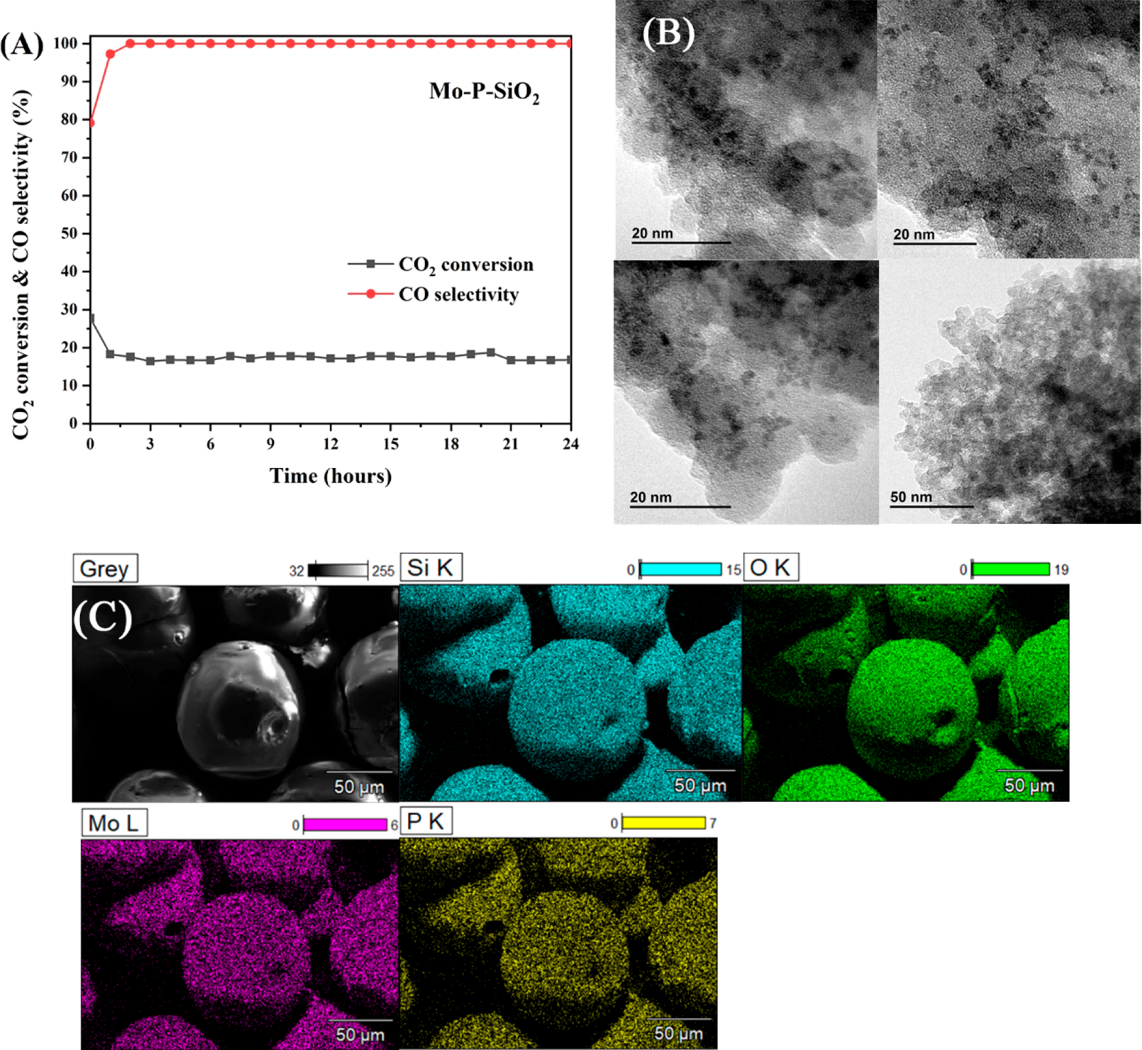
(A) Stability test at 550 °C, WHSV of 12 000 mL g^–1^ h^–1^ with a H_2_/CO_2_ ratio of 4:1 for Mo–P–SiO_2_. (B) TEM micrographs of Mo–P–SiO_2_. (C) EDX micrographs of Mo–P–SiO_2_.

As the results show in Figure 4A, the CO_2_ conversion declined from 27% to 18% in the first 2 h of testing, and the CO selectivity increased from 80% to 97% in the first hour and reached at 100% at 2 h. After 2 h, both the CO_2_ conversion and CO selectivity remained stable in the remaining 22 h, indicating carbon deposition occurs initially, after which catalytic activity is stabilized. Overall, our catalysts exhibit a stable performance once the steady state is reached showcasing full selectivity to CO at intermediate temperatures where CO_2_ methanation is typically an issue.^47^

TEM characterization was used to study the nanostructure of as synthesized Mo–P–SiO_2_ (Figure 4B). Spherical MoP nanoparticles can be seen in Figure 4B, similar to MoP/SiO_2_ catalysts reported previously.^13^ The corresponding element mappings of Mo–P–SiO_2_ shown in Figure 4C demonstrate that the elements of Mo and P are uniformly co-located on the entire nanoparticles of the SiO_2_ support. For the silicon-supported MoP catalysts, our previous works show that the catalyst synthesized in this same technique yields a mixture of phosphate and phosphide,^20^ which might be the reason that the MoP peaks have not been observed in the XRD pattern. Since we have proven in the TPR section that the reduction temperature we used in synthesis is suitable for silicon-supported MoP production, and spherical MoP nanoparticles detected in Figure 4B are similar to the MoP/SiO_2_ catalysts reported in previous work,^13^ the catalysts we synthesized in here are likely to be the mixture of phosphide/phosphate.

Our results show that supported Mo–P catalysts are robust materials that can run satisfactorily for continuous operations displaying complete RWGS selectivity. The suppression of the Sabatier reaction is particularly significant for the efficient use of hydrogen; for a net CO_2_ consuming RWGS process, H_2_ should have a low carbon footprint and currently green H_2_ is expensive as well.^3^ Moreover, the complete RWGS selectivity across the full range of temperatures and conversions studied herein make it possible to explore tandem catalysis schemes where MoP catalysts producing CO could be coupled with CO consuming Fischer–Tropsch active catalysts. This area of tandem catalysis for CO_2_ utilization has gathered considerable interest and requires the development of fully selective RWGS catalysts.^48^Table 3 presents the comparative performance of MoP catalysts in this work with prior investigations. Although MoP has been reported to be used in some reactions such as alcohol synthesis, to the best of our knowledge, no other paper has reported MoP as a catalyst for the RWGS reaction. Therefore, we have compared the performance to molybdenum carbides as well as our recent work on nickel phosphide catalysts (Table 3). We have previously shown the activity of nickel phosphide toward the RWGS reaction, and it exhibited higher CO_2_ activity at the same temperature as MoP-SiO_2_ reported here.^49^ However, unlike the MoP catalysts presented herein, nickel phosphides are also active for methanation, especially at the low temperature range (300–600 °C). We have also studied previously the performance of molybdenum carbides toward the RWGS reaction. The β-Mo_2_C shows higher CO_2_ activity than the MoP catalyst in this work, and with the addition of Cs or Cu, the CO selectivity reached 95–98%. However, for the 0.25 g Mo_2_C catalyst used in our previous test, it contains 100 wt % Mo_2_C (or 99 wt % Mo_2_C for Cu–Mo_2_C and Cs–Mo_2_C) in the catalyst. For the 0.25 g MoP catalysts used in this work, there is only 15 wt % MoP in the catalyst. Therefore, in terms of the mass activity, MoP is still a promising catalyst for the RWGS reaction.^50^

**Table 3:** Catalyst Performance Comparison with Materials Reported in the Literature

catalysts	temp (°C)	H_2_/CO_2_ ratio	CO_2_ conversion (%)	CO selectivity (%)	WHSV (mL/g_cal_ h)	ref
1%NiCo@SiO_2_	500	4	50	47	15000	(51)
β-Mo_2_C	550	4	60	85		
Cu–Mo_2_C	550	4	58	95	12000	(50)
Cs–Mo_2_C	550	4	56	98		
Ni_2_P–-SiO_2_	550	4	43	79	12000	(49)
Mo–P–SiO_2_	550	4	18	100	12000	this work

## 4 Conclusions

In this work, we have synthesized supported MoP catalysts to investigate their activity in the RWGS reaction, which demands a stable and fully selective catalyst capable of operating at increased temperatures. Silica, alumina, and ceria-alumina supported MoP catalysts are all shown to be active for the RWGS reaction and demonstrate a complete suppression of the methanation side reaction. Mo–P–SiO_2_ showed limited deactivation in the first 2 h of the test due to carbon deposition, followed by stable performance for 22 h on stream. This high selectivity of MoP catalysts to CO is a significant advancement toward developing robust RWGS catalysts that make efficient use of green hydrogen, which is needed to develop net CO_2_ consuming processes. Moreover, MoP catalysts provide a step forward in developing tandem catalysts that can synthesize coupled carbon products from CO. The discovery of new catalysts for RWGS opens up opportunities for chemical CO_2_ recycling which are urgently needed in the context of a circular economy.

## Supporting Information Available

Detailed explanations of the experimental protocols as well as data from BET measurements and carbon balance calculations (PDF)

## References

[ref1] Tauseef Hassan S.; Xia E.; Lee C. C. Mitigation pathways impact of climate change and improving sustainable development: The roles of natural resources, income, and CO2 emission. Energy Environ. 2021, 32, 338–363. 10.1177/0958305X20932550.

[ref2] Guharoy U.; Ramirez Reina T.; Gu S.; Cai Q. Mechanistic insights into selective CO2 Conversion via RWGS on Transition Metal Phosphides: A DFT Study. J. Phys. Chem. C 2019, 123, 22918–22931. 10.1021/acs.jpcc.9b04122.

[ref3] Bown R. M.; Joyce M.; Zhang Q.; Reina T. R.; Duyar M. S. Identifying Commercial Opportunities for the Reverse Water Gas Shift Reaction. Energy Technol. 2021, 9, 28–31. 10.1002/ente.202100554.

[ref4] Zhang Q.; Pastor-Pérez L.; Gu S.; Reina T. R. Transition metal carbides (TMCS) catalysts for gas phase CO2 upgrading reactions: A comprehensive overview. Catalysts 2020, 10, 1–23. 10.3390/catal10090955.

[ref5] Porosoff M. D.; Yan B.; Chen J. G. Catalytic reduction of CO2 by H2 for synthesis of CO, methanol and hydrocarbons: Challenges and opportunities. Energy Environ. Sci. 2016, 9, 62–73. 10.1039/C5EE02657A.

[ref6] Zhou G.; Xie F.; Deng L.; Zhang G.; Xie H. Supported mesoporous Cu/CeO2-δ catalyst for CO2 reverse water–gas shift reaction to syngas. Int. J. Hydrogen Energy 2020, 45, 11380–11393. 10.1016/j.ijhydene.2020.02.058.

[ref7] Kobayashi D.; Kobayashi H.; Kusada K.; Yamamoto T.; Toriyama T.; Matsumura S.; Kawaguchi S.; Kubota Y.; Haneda M.; Aspera S. M.; Nakanishi H.; Arai S.; Kitagawa H. Boosting reverse water-gas shift reaction activity of Pt nanoparticles through light doping of W. J. Mater. Chem. A 2021, 9, 15613–15617. 10.1039/D1TA03480D.

[ref8] Zhu M.; Ge Q.; Zhu X. Catalytic Reduction of CO2 to CO via Reverse Water Gas Shift Reaction: Recent Advances in the Design of Active and Selective Supported Metal Catalysts. Trans. Tianjin Univ. 2020, 26, 172–187. 10.1007/s12209-020-00246-8.

[ref9] Tang R.; Zhu Z.; Li C.; Xiao M.; Wu Z.; Zhang D.; Zhang C.; Xiao Y.; Chu M.; Genest A.; Rupprechter G.; Zhang L.; Zhang X.; He L. Ru-Catalyzed Reverse Water Gas Shift Reaction with Near-Unity Selectivity and Superior Stability. ACS Mater. Lett. 2021, 3, 1652–1659. 10.1021/acsmaterialslett.1c00523. 34901871 PMC8653414

[ref10] Levy R. B.; Boudart M. Platinum-like behavior of tungsten carbide in surface catalysis. Science (80-.) 1973, 181, 547–549. 10.1126/science.181.4099.547. 17777803

[ref11] Stinner C.; Prins R.; Weber T. Binary and ternary transition-metal phosphides as HDN catalysts. J. Catal. 2001, 202, 187–194. 10.1006/jcat.2001.3283.

[ref12] Sun M.; Liu H.; Qu J.; Li J. Earth-Rich Transition Metal Phosphide for Energy Conversion and Storage. Adv. Energy Mater. 2016, 6, 1600087 10.1002/aenm.201600087.

[ref13] Duyar M. S.; Tsai C.; Snider J. L.; Singh J. A.; Gallo A.; Yoo J. S.; Jaramillo T. F.; et al. A Highly Active Molybdenum Phosphide Catalyst for Methanol Synthesis from CO and CO2. Angew. Chemie - Int. Ed. 2018, 57, 15045–15050. 10.1002/anie.201806583. 30134041

[ref14] Duyar M. S.; Gallo A.; Regli S. K.; Snider J. L.; Singh J. A.; Valle E.; Jaramillo T. F. Understanding selectivity in co2 hydrogenation to methanol for mop nanoparticle catalysts using in situ techniques. Catalysts 2021, 11, 1–18. 10.3390/catal11010143.

[ref15] Yao Z.; Lai Z.; Zhang X.; Peng F.; Yu H.; Wang H. Structural stability and mutual transformations of molybdenum carbide, nitride and phosphide. Mater. Res. Bull. 2011, 46, 1938–1941. 10.1016/j.materresbull.2011.07.023.

[ref16] Jurković D. L.; Pohar A.; Dasireddy V. D. B. C.; Likozar B. Effect of Copper-based Catalyst Support on Reverse Water-Gas Shift Reaction (RWGS) Activity for CO2 Reduction. Chem. Eng. Technol. 2017, 40, 973–980. 10.1002/ceat.201600594.

[ref17] Pastor-Pérez L.; Shah M.; Le Saché E.; Reina T. R. Improving Fe/Al 2 O 3 catalysts for the reverse water-gas shift reaction: On the effect of cs as activity/selectivity promoter. Catalysts 2018, 8, 608 10.3390/catal8120608.

[ref18] Yang L.; Pastor-Pérez L.; Gu S.; Sepúlveda-Escribano A.; Reina T. R. Highly efficient Ni/CeO2-Al2O3catalysts for CO2upgrading via reverse water-gas shift: Effect of selected transition metal promoters. Appl. Catal. B Environ. 2018, 232, 464–471. 10.1016/j.apcatb.2018.03.091.

[ref19] Reina T. R.; Moreno A. Á.; Ivanova S.; Odriozola J. A.; Centeno M. A. Influence of Vanadium or Cobalt Oxides on the CO Oxidation Behavior of Au/MO x/CeO 2-Al 2O 3 Systems. ChemCatChem. 2012, 4, 512–520. 10.1002/cctc.201100373.

[ref20] ten Have I. C.; Valle E.; Gallo A.; Snider J. L.; Duyar M. S.; Jaramillo T. F. Development of Molybdenum Phosphide Catalysts for Higher Alcohol Synthesis from Syngas by Exploiting Support and Promoter Effects. Energy Technol. 2019, 7, 1–14. 10.1002/ente.201801102.

[ref21] Wu Z. G.; Jia Y. R.; Wang J.; Guo Y.; Gao J. F. Core-shell SiO2/Ag composite spheres: Synthesis, characterization and photocatalytic properties. Mater. Sci. Polym. 2016, 34, 806–810. 10.1515/msp-2016-0121.

[ref22] Iino A.; Cho A.; Takagaki A.; Kikuchi R.; Ted Oyama S. Kinetic studies of hydrodeoxygenation of 2-methyltetrahydrofuran on a Ni2P/SiO2 catalyst at medium pressure. J. Catal. 2014, 311, 17–27. 10.1016/j.jcat.2013.11.002.

[ref23] Pastor-Pérez L. L.; Baibars F.; Le Sache E.; Arellano-Garcia H.; Gu S.; Reina T. R. CO2 valorisation via Reverse Water-Gas Shift reaction using advanced Cs doped Fe-Cu/Al2O3 catalysts. J. CO2 Util. 2017, 21, 423–428. 10.1016/j.jcou.2017.08.009.

[ref24] Deliy I.; Shamanaev I.; Aleksandrov P.; Gerasimov E.; Pakharukova V.; Kodenev E.; Yakovlev I.; Lapina O.; Bukhtiyarova G. Support effect on the performance of Ni2P catalysts in the hydrodeoxygenation of methyl palmitate. Catalysts 2018, 8, 515 10.3390/catal8110515.

[ref25] Yang Z. M.; Huang G. F.; Huang W. Q.; Wei J. M.; Yan X. G.; Liu Y. Y.; Pan A. Novel Ag3PO4/CeO2 composite with high efficiency and stability for photocatalytic applications. J. Mater. Chem. A 2014, 2, 1750–1756. 10.1039/C3TA14286H.

[ref26] Ma R.; Jahurul Islam M.; Amaranatha Reddy D.; Kim T. K. Transformation of CeO2 into a mixed phase CeO2/Ce2O3 nanohybrid by liquid phase pulsed laser ablation for enhanced photocatalytic activity through Z-scheme pattern. Ceram. Int. 2016, 42, 18495–18502. 10.1016/j.ceramint.2016.08.186.

[ref27] Wan C.; Regmi Y. N.; Leonard B. M. Multiple Phases of Molybdenum Carbide as Electrocatalysts for the Hydrogen Evolution Reaction. Angew. Chem. 2014, 126, 6525–6528. 10.1002/ange.201402998. 24827779

[ref28] Cheekatamarla P. K.; Thomson W. J. Poisoning effect of thiophene on the catalytic activity of molybdenum carbide during tri-methyl pentane reforming for hydrogen generation. Appl. Catal. A Gen. 2005, 287, 176–182. 10.1016/j.apcata.2005.03.043.

[ref29] Oshikawa K.; Nagai M.; Omi S. Characterization of Molybdenum Carbides for Methane Reforming by TPR, XRD, and XPS 2001, 9124–9131.

[ref30] Ma Y.; Guan G.; Hao X.; Zuo Z.; Huang W.; Phanthong P.; Abudula A. Highly-efficient steam reforming of methanol over copper modified molybdenum carbide. RSC Adv. 2014, 4, 44175–44184. 10.1039/C4RA05673F.

[ref31] Liu C.; Lin M.; Jiang D.; Fang K.; Sun Y. Preparation of promoted molybdenum carbides nanowire for CO hydrogenation. Catal. Lett. 2014, 144, 567–573. 10.1007/s10562-013-1163-7.

[ref32] Choi J. G.; Thompson L. T. XPS study of as-prepared and reduced molybdenum oxides. Appl. Surf. Sci. 1996, 93, 143–149. 10.1016/0169-4332(95)00317-7.

[ref33] Yang L.; Zhou W.; Hou D.; Zhou K.; Li G.; Tang Z.; Chen S. Porous metallic MoO _2_ -supported MoS _2_ nanosheets for enhanced electrocatalytic activity in the hydrogen evolution reaction. Nanoscale 2015, 7, 5203–5208. 10.1039/C4NR06754A. 25700339

[ref34] Phillips D. C.; Sawhill S. J.; Self R.; Bussell M. E. Synthesis, characterization, and hydrodesulfurization properties of silica-supported molybdenum phosphide catalysts. J. Catal. 2002, 207, 266–273. 10.1006/jcat.2002.3524.

[ref35] Xie S.; Gou J. Facile synthesis of Ni2P/Ni12P5 composite as long-life electrode material for hybrid supercapacitor. J. Alloys Compd. 2017, 713, 10–17. 10.1016/j.jallcom.2017.04.170.

[ref36] Wang Z.; Wang S.; Ma L.; Guo Y.; Sun J.; Zhang N.; Jiang R. Water-Induced Formation of Ni2P–Ni12P5 Interfaces with Superior Electrocatalytic Activity toward Hydrogen Evolution Reaction. Small 2021, 17, 1–9. 10.1002/smll.202006770. 33470529

[ref37] Shi H.; Chen J.; Yang Y.; Tian S. Catalytic deoxygenation of methyl laurate as a model compound to hydrocarbons on nickel phosphide catalysts: Remarkable support effect. Fuel Process. Technol. 2014, 118, 161–170. 10.1016/j.fuproc.2013.08.010.

[ref38] Li X.; Zhang Y.; Wang A.; Wang Y.; Hu Y. Influence of TiO2 and CeO2 on the hydrogenation activity of bulk Ni2P. Catal. Commun. 2010, 11, 1129–1132. 10.1016/j.catcom.2010.06.006.

[ref39] Zaman S. F.; Smith K. J. Synthesis gas conversion over a Rh-K-MoP/SiO2 catalyst. Catal. Today 2011, 171, 266–274. 10.1016/j.cattod.2011.02.017.

[ref40] Clark P. A.; Oyama S. T. Alumina-supported molybdenum phosphide hydroprocessing catalysts. J. Catal. 2003, 218, 78–87. 10.1016/S0021-9517(03)00086-1.

[ref41] Großmann K.; Dellermann T.; Dillig M.; Karl J. Coking behavior of nickel and a rhodium based catalyst used in steam reforming for power-to-gas applications. Int. J. Hydrogen Energy 2017, 42, 11150–11158. 10.1016/j.ijhydene.2017.02.073.

[ref42] Schulz L. A.; Kahle L. C.; Delgado K. H.; Schunk S. A.; Jentys A.; Deutschmann O.; Lercher J. A. On the coke deposition in dry reforming of methane at elevated pressures. Appl. Catal. A Gen. 2015, 504, 599–607. 10.1016/j.apcata.2015.03.002.

[ref43] Therdthianwong S.; Summaprasit N. Synthesis Gas Production from CH 4 Reforming with CO 2 over Pd/Al 2 O 3 Promoted with CeO 2. Asian J. Energy Environ. 2002, 3, 1–25.

[ref44] Erdőhelyi A. Catalytic reaction of carbon dioxide with methane on supported noble metal catalysts. Catalysts 2021, 11, 1–30. 10.3390/catal11020159.

[ref45] Marafi A.; Hauser A.; Stanislaus A. Deactivation patterns of Mo/Al2O3, Ni-Mo/Al2O3 and Ni-MoP/Al2O3 catalysts in atmospheric residue hydrodesulphurization. Catal. Today 2007, 125, 192–202. 10.1016/j.cattod.2007.03.060.

[ref46] Shamsi A.; Baltrus J. P.; Spivey J. J. Characterization of coke deposited on Pt/alumina catalyst during reforming of liquid hydrocarbons. Appl. Catal. A Gen. 2005, 293, 145–152. 10.1016/j.apcata.2005.07.002.

[ref47] Pastor-Pérez L.; Patel V.; Le Saché E.; Reina T. R. CO2 methanation in the presence of methane: Catalysts design and effect of methane concentration in the reaction mixture. J. Energy Inst. 2020, 93, 415–424. 10.1016/j.joei.2019.01.015.

[ref48] Ma Z.; Porosoff M. D. Development of Tandem Catalysts for CO 2 Hydrogenation to Olefins. ACS Catal. 2019, 9, 2639–2656. 10.1021/acscatal.8b05060.

[ref49] Zhang Q.; Villora-pico J. J.; Joyce M.; Sepúlveda-escribano A. Ni-Phosphide catalysts as versatile systems for gas-phase CO 2 conversion : Impact of the support and evidences of structure-sensitivity. Fuel 2022, 323, 1–12. 10.1016/j.fuel.2022.124301.

[ref50] Zhang Q.; Pastor-pérez L.; Jin W.; Gu S.; Reina T. r. Understanding the promoter effect of Cu and Cs over highly effective β-Mo2C catalysts for the reverse water-gas shift reaction. Appl. Catal. B Environ. 2019, 244, 889–898. 10.1016/j.apcatb.2018.12.023.

[ref51] Price C. A. H.; Pastor-Perez L.; Reina T. R.; Liu J. Yolk-Shell structured NiCo@SiO2 nanoreactor for CO2 upgrading via reverse water-gas shift reaction. Catal. Today 2022, 383, 358–367. 10.1016/j.cattod.2020.09.018.

