# Supplementary material for: CO2 Conversion via Reverse Water Gas Shift Reaction Using Fully Selective Mo–P Multicomponent Catalysts
Source: Ind Eng Chem Res. 2022 Aug 19;61(34):12857–65. doi: 10.1021/acs.iecr.2c00305 (PMC9437872; doi:10.1021/acs.iecr.2c00305)
Supplement: Supplementary file 1 — ie2c00305_si_001.pdf [file ie2c00305_si_001.pdf]

CO_2 conversion via RWGS using fully selective Mo-P multicomponent catalysts

Qi Zhang, Matthew Bown, Laura Pastor-Pérez, Melis S. Duyar, Tomas R. Reina**

Department of Chemical and Process Engineering, University of Surrey, Guildford, GU2 7XH, United Kingdom

Supporting Information

Contents

1. Experimental Section 2
1.1 Catalysts Characterization 2
1.2 Catalytic Testing 3
2. Textural properties 3
3. Carbon balance calculation 4
Reference: 5

1. Experimental Section

1.1 Catalysts Characterization

X-ray photoelectron spectroscopy (XPS, K-ALPHA, Thermo Scientific) was carried out to analyse the surface chemistry of the prepared materials. The catalysts were exposed to air prior to analysis however, they had been passivated during synthesis to minimise oxidation. All spectra were collected using Al-K radiation (1486.6 eV), monochromatized by a twin crystal monochromator, yielding a focused X-ray spot (elliptical in shape with a major axis length of 400 μm) at 3 mA × 12 kV. The alpha hemispherical analyser was operated in the constant energy mode with survey scan pass energies of 200 eV to measure the whole energy band and 50 eV in a narrow scan to selectively measure the particular elements. XPS data were analysed with Avantage software. The C 1s core level located at 284.6 eV was used as reference binding energy. The experimental backgrounds were corrected by the smart background function. The system flood gun that provides low energy electrons and low energy argon ions from a single source was used to achieve charge compensation.

X-Ray Diffraction (XRD) analysis was used to detect the crystal structures of the prepared catalysts with an X'Pert Pro PANalytical at room temperature using Cu-K$^{\circ}$ (40mA, 45kV) over a 2 theta range of 10°-80°.

The hydrogen consumption of the catalyst precursors was determined by H_2-Temperature programmed reduction (TPR). The experiment was conducted in a vertical fixed bed quartz reactor under the mixture of 5 mL/min H_2 and 20 mL/min Ar. The right amount of quartz wool was placed into the reactor and 50 mg of catalyst precursor was loaded on the quartz wool, and then heated up to 920 $^{\circ}$C at a ramp of 10 $^{\circ}$C/min. The hydrogen consumption and water production were recorded by an online mass spectrometer (Pfeiffer, OmniStar GSD 301).

The N_2 adsorption at −196 °C with an AUTOSORB-6 equipment (QUANTACHROME INSTRUMENTS) was carried out to determine the surface area by using the Brunauer–Emmett–Teller (BET) equation. The Barret–Joyner–Halenda (BJH) method was used to estimate the average pore size and pore volume. Samples were previously out-gassed at 250 °C under vacuum for 4 h.

Temperature-programmed oxidation (TPO) experiments were performed to quantify the amount of carbon deposited on the used catalysts. A stream of 3% O_2 in He (40 mL/min) was passed over the sample while the temperature was increased from room temperature to 920 ^{o}C at 10 ^{o}C/min. The effluent gas was analysed using an online mass spectrometer (Pfeiffer, OmniStar GSD 301), and mass 44 (CO_2) was recorded. The CO_2 signal was calibrated so that the TPO peak area could be converted to the amount of carbon deposited.

Thermogravimetric analysis (TGA) was conducted on the post reaction catalysts in an SDT Q600 V8.3 Instrument from TA Instruments. A flow of 100 mL/min of air was used, while the temperature was increased from room temperature to 900 ◦C at 10◦C/min.

1.2 Catalytic Testing

The RWGS reactions were conducted and recorded in a vertical continuous fixed bed reactor coupled to an ABB AO2020 Advanced Optima Process Gas Analyser that was used for the on-line analysis of reactants and products (CO and CH_4). 0.25 g of catalyst was loaded on the quartz wool in the middle of a 7mm inner diameter quartz reactor. The sample was heated in flowing N_2 from room temperature to 400 ºC. Then, the feed gas was replaced by a mixture of H_2:CO_2 = 4:1 at a constant weight-hourly space velocity (WHSV) of 12000 mL g^{-1} h^{-1}. For all the studied catalysts, tests were evaluated within a temperature range of 400 to 750 °C. At each temperature, the gas products were detected and analysed after reaching steady state. Stability tests were conducted at a space velocity of 12000 mL g^{-1} h^{-1} with a H_2:CO_2 ratio of 4:1 at 550 °C for 24 h. The continuous temperature programmed RWGS reactions were conducted within a temperature range of 300 to 750 °C using the mass spectrum (Pfeiffer, OmniStar GSD 301) for product analysis at a space velocity of 12000 mL g^{-1} h^{-1} with a H_2:CO_2 ratio of 4:1.

Performance of the catalysts are reported in terms of CO_2 conversion (*eq. (3)*), CO selectivity (*eq. (4)*) and CH_4 selectivity (*eq. (5)*). The relative experimental error in CO_2 conversion and CO/CH_4 selectivity in this work was given within ± 0.5 % [2]. Where n_{CO_2in} is the initial molar flow rate (kmol/min) of CO_2 in the reactant mixture and $n_{COout}, n_{CH_4out}, n_{CO_2out}$ are the outlet molar flow rates in the product stream of CO, CH_4 and CO_2 respectively.

$$CO_2\ conversion\ (\%) = \frac{n_{CO_2in} - n_{CO_2out}}{n_{CO_2in}} \cdot 100 \qquad eq.(3)$$

$$CO\ selectivity\ (\%) = \frac{n_{COout}}{n_{CO_2in} - n_{CO_2out}} \cdot 100 \qquad eq.(4)$$

$$CH_4\ selectivity\ (\%) = \frac{n_{CH_4out}}{n_{CO_2in} - n_{CO_2out}} \cdot 100 \qquad eq.(5)$$

2. Textural properties

The BET surface area, pore volume and pore size of the studied samples are summarized in Table S1. The surface area of MoP/SiO_2 is 235m^2/g, twice the value of MoP/CeAl catalyst (104m^2/g) and MoP/Al_2O_3 catalyst (147m^2/g). In addition, MoP/SiO_2 catalyst has nearly twice as big pore volume as MoP/Al_2O_3 and MoP/CeAl. While there are some differences in surface area the average pore size is similar, MoP particle size distribution is expected to be similar via the impregnation method used.

Table S1 Textual properties of studied samples.

Catalysts	Surface area (m^2/g)	Pore volume (cm^3/g)	Pore size (nm)

Mo-P-SiO_2	235	0.5	8.5
Mo-P-Al_2O_3	147	0.33	8.7
Mo-P-CeAl	104	0.23	9.1

a. calculated by the BET equation.

b. Pore volumes calculated from the N2 desorption at a relative pressure of 0.96.

c. BJH desorption average pore diameter.

3. Carbon balance calculation

Since the carbon balance towards synthesized catalysts did not reach at 100%. The calculation of missing carbon from RWGS tests and the corresponding carbon deposition have been listed below. The method using for the missing carbon calculation towards Mo-P-SiO_2 was shown in table S2 as an example:

Table S2. Mo-P-SiO_2 performance for RWGS reaction.

Temperature 450-750^{0}C and molar ratio 4:1														
	%vol						molar flow (mmol/min)							
Bypass	CO	CH_4	CO_2	H_2	Flow (mL/min)		CO	CH_4	CO_2	H_2		CO_2 conversion	CO selecitivity	CO2in-CO2out-COout (mmol/min)
	0	0	10.73	41.02	49.98		0	0	0.23941	91.5259				
	0	0	11.64	40.99	50.74		0	0	0.26367	92.8497				
	0	0	11.69	41.2	51.15		0	0	0.26694	94.0795				
	0	0	11.66	41.2	51.17		0	0	0.26636	94.1163				
	0	0	11.69	41.95	51.39		0	0	0.26819	96.2415				
	0	0	11.66	42.14	51.26		0	0	0.26683	0.96433				
							0	0	0.26091	93.7626				
Temperature														
450.00	0.67	0	10.97	41.71	50.21		0.01502	0	0.24589	0.93494		5.756394	99.9929852	1.05357E-06
500.00	1.48	0	10.19	40.93	49.92		0.03298	0	0.22709	0.91215		12.96302	97.5180684	0.000839446
550.00	2.43	0	9.2	39.96	50.31		0.05458	0	0.20663	0.89749		20.8051	100.541586	-0.000293991
600.00	3.72	0	7.96	38.73	48.76		0.08098	0	0.17327	0.84307		33.59026	92.3949796	0.006665161
650.00	5.22	0	6.29	37.32	48.04		0.11195	0	0.1349	0.80038		48.29784	88.8384228	0.014065339
700.00	6.6	0	4.71	36.09	48		0.14143	0	0.10093	0.77336		61.31726	88.4010559	0.018556589
750.00	7.4	0	3.89	35.24	46.42		0.15335	0	0.08061	0.73029		69.10346	85.0534782	0.026948643
														0.067075179

The molar flow of missing carbon was obtained according to the following equation where $n_{CO_2 in}$ is the initial molar flow rate (kmol/min) of CO_2 in the reactant mixture and n_{COout}, $n_{CO_2 out}$ are the outlet molar flow rates in the product stream of CO and CO_2 respectively, i represents the temperatures used in the RWGS test:

$$M = \sum_i (n_{CO_2 in} - n_{CO_2 out} - n_{COout})i \times 60min$$

Since the catalyst was tested for 1 hour at each temperature, the molar flow of missing carbon is 4.026mmol (the sum of the right column in table 1: 0.0671mmol/min × 60min), the mass of missing carbon is 48.312mg. According to the TGA results, the carbon deposition on Mo-P-SiO_2 catalyst is 36.1mg. Therefore, around 75% of the missing carbon became the coke formation deposited on the surface of the Mo-P-SiO_2 catalysts. Indeed, the data indicate that there are small amounts of gas phase products (other than CO) that is unable to account for.

Same method has been used for the calculation for Mo-P-Al_2O_3 and Mo-P-CeAl. The missing carbon during the RWGS test towards Mo-P-Al_2O_3 and Mo-P-CeAl are around 66.3mg and 56.3mg respectively.

Reference:

[1] ten Have, I. C., Valle, E., Gallo, A., Snider, J. L., Duyar, M. S., & Jaramillo, T. F. (2019). Development of Molybdenum Phosphide Catalysts for Higher Alcohol Synthesis from Syngas by Exploiting Support and Promoter Effects. Energy Technology, 7(5), 1–14.

[2] Yang, Z. M., Huang, G. F., Huang, W. Q., Wei, J. M., Yan, X. G., Liu, Y. Y., ... & Pan, A. (2014). Novel Ag_3PO_4/CeO_2 composite with high efficiency and stability for photocatalytic applications. Journal of Materials Chemistry A, 2(6), 1750-1756.
